# A feasibility trial of advance care planning for patients with terminal cancer in primary hospitals

**DOI:** 10.1186/s12904-025-01920-1

**Published:** 2025-11-03

**Authors:** Yi Li, Siyan Liang, Liyun Huang, Zhijun Ding

**Affiliations:** 1 Xiaolan Clinical Institute of Shantou University Medical College, Zhongshan, China; 2 Department of Medical Oncology, Xiaolan People’s Hospital of ZhongShan (The Fifth People’s Hospital of ZhongShan), Zhongshan, China; 3 Department of Orthopedics, Jiangmen Hospital of Traditional Chinese Medicine Affiliated to Jinan University, Jiangmen, China

**Keywords:** Advance care planning, Primary hospitals, Patients with terminal cancer, Palliative care.

## Abstract

**Objective:**

To investigate the effects of advance care planning (ACP) on patients with terminal cancer in primary hospitals.

**Methods:**

Convenience sampling was used to select 60 patients with terminal cancer and their primary caregivers from primary hospitals. The control group received routine care, while the intervention group received routine care plus an ACP-based care intervention. Patients and their primary caregivers’ anxiety and depression levels were assessed before and after the intervention. We compared several outcomes between the ACP intervention and routine care control group, including quality of life scores (EORTC QLQ-C30), frequency of intensive care unit (ICU) admissions, length of hospital stay within 30 days before death, and direct medical costs.

**Results:**

Two weeks post-intervention, caregivers’ anxiety and depression were significantly lower in the ACP group than in the control group (*P* < 0.05). The ACP group also showed significant improvements in quality of life, including role functioning, pain, nausea/vomiting, dyspnea, constipation, diarrhea. At 1 month post-intervention, both patients and caregivers had significantly reduced anxiety/depression in the ACP group (*P* < 0.05). All quality-of-life dimensions significantly improved with ACP (*P* < 0.05). There were also significant reductions in ICU admission frequency, length of hospital stay within 30 days before death, and direct medical costs in the ACP group relative to the control group (*P* < 0.05).

**Conclusion:**

ACP intervention can be successfully implemented for patients with terminal cancer in primary hospitals when clinicians receive structured training and standardized documentation and family engagement protocols are followed. Furthermore, the findings suggest that ACP may help reduce anxiety and depression in patients and their caregivers, improve patients’ quality of life, and has the potential to decrease ICU admissions, hospital stay length, and direct medical costs.

## Introduction

The Global Cancer Statistics 2020 report showed that China had 4.568 million new cancer cases, representing 23.7% of the global total. Cancer deaths in China reached 3.003 million, accounting for 30.2% of the worldwide total [1, 2]. Influenced by Confucian culture, most families focus on prolonging the lives of terminally ill patients, sometimes overlooking the patients’ own wishes. This leads to overtreatment, which increases patient suffering and reduces their quality of life [3, 4]. To address this issue, in 2017, the National Health Commission of China issued the Palliative Care Practice Guidelines (Trial Implementation) [5]. As a crucial measure for implementing patient rights respect outlined in these guidelines, Advance Care Planning (ACP) urgently requires promotion [6]. In 2023, China’s National Health Commission and five other departments issued a policy aimed at expanding the provision of palliative care and launching a capacity-building initiative to improve these services [7].

ACP is a new concept that builds on the principles of palliative care and has emerged from palliative care research. ACP refers to a process in which patients, while still possessing decision-making capacity, express their future treatment and care preferences after being informed about their prognosis and available medical options [8]. The concept of ACP was first proposed as early as 1993. Since then, international research on ACP has increased annually, gaining recognition and promotion in multiple countries worldwide. Although Japan has not yet passed legislation specifically addressing ACP, the proportion of adults participating in ACP discussions rose significantly from 20% in 2006 to 42% in 2014 [9]. During the COVID-19 pandemic, Ye et al. conducted a retrospective study across 15 U.S. nursing homes and found that proactive ACP conversations between healthcare providers and elderly patients or their families facilitated clearer documentation of medical preferences. This approach effectively reduced unnecessary hospitalizations and conserved medical resources during the crisis [10]. Shen et al. [11] explored preferred ACP communication approaches among terminal cancer patients in Latin America, revealing their strong preference for family-centered, physician-guided discussions.

The core purposes of ACP are to reduce psychological distress caused by uncertainty among patients and families by enhancing predictability and sense of control over future medical care, and to ensure that medical care aligns with patients’ personal values and life goals, thereby avoiding futile or non-beneficial treatments that they would not desire and that may reduce their quality of life [3, 12]. Numerous studies have shown that ACP respects patient autonomy, preserves patient dignity, improves quality of life, reduces levels of anxiety and depression in both patients and their families, and decreases overtreatment [4, 8, 10, 13]. Compared with research in developed countries, domestic ACP studies reveal notable structural imbalances in research focus and methodology. Current evidence predominantly focuses on descriptive investigations of knowledge and attitudes [14], while higher-level evidence regarding intervention design and outcome evaluation remains scarce. Furthermore, localized ACP implementation models tailored to primary hospitals are insufficiently developed, limiting their practical application in these settings [15, 16]. To address this gap, it is essential to deepen research on ACP within primary hospital contexts [17]. In this study, we aim to develop a culturally adapted ACP protocol suitable for primary hospitals in China and comprehensively evaluate its effectiveness across multiple dimensions: psychological outcomes (anxiety and depression), core outcomes (quality of life), health system indicators (medical expenditures), and quality-of-care process measures (ICU transfers and length of hospital stay). The methodology and findings are detailed below.

## Participants and methods

### General information

Convenience sampling was used to select 60 terminal cancer patients and their primary caregivers. These participants, admitted to the oncology department of the researchers’ institution from April 2022 to July 2023, served as study subjects. The enrolled participants were then randomly assigned to either the control group or the intervention group (30 participants each) using a computer-generated random number table. All participants had received health education on ACP and demonstrated understanding of its concepts.

Patient inclusion criteria were as follows: (1) Diagnosis of malignant tumors confirmed by biopsy or intraoperative pathology; (2) Meeting the 2011 Royal College of General Practitioners Gold Standards Framework guidelines for terminal stage diagnosis, requiring at least two of the following: metastasis, cachexia, pleural or peritoneal effusion, severe pain, increased intracranial pressure, respiratory dysfunction, or life expectancy under 6 months; (3) No history of mental illness or cognitive impairment and possessing intact self-expression; (4) Awareness of their condition; (5) Age over 18 years; (6) Residency in Xiaolan Town; (7) Provided informed consent and agreed to participate.

Primary caregiver inclusion criteria were as follows: (1) Patient’s spouse, parent, child, or other relative; (2) Responsible for the patient’s daily care for at least 72 continuous hours [18] and designated by the patient as the primary caregiver; (3) Age over 18 years; (4) Normal cognitive function and capable of completing questionnaires; (5) Provided informed consent and agreed to participate.

Exclusion criteria were as follows: (1) Patients with cognitive or comprehension impairments; (2) Patients or caregivers with communication or comprehension barriers; (3) Individuals unable to tolerate discussions lasting longer than 60 min; (4) Patients or caregivers who refused to participate.

This study was approved by the hospital ethics committee (Ethics Number: ZSXL-LL2021-030). All participants were fully informed and signed an informed consent form.

### Methods

The intervention methods for the control group included the implementation of routine care for patients with cancer, consisting of the following: (1) Symptom management: continuous monitoring of disease progression and providing targeted treatment and care for symptoms such as pain, coughing, fatigue, diarrhea, constipation, dyspnea, nausea, vomiting, and insomnia; (2) Psychological care: assessment of the patient’s psychological state; providing care, comfort, and encouragement; and helping them to have a positive and optimistic attitude toward treatment and life; (3) Discharge guidance: advising patients to take medications on time, exercise moderately, ensure adequate rest, and seek medical attention in case of discomfort; (4) Follow-up: Telephone follow-ups were conducted 2 weeks after discharge, and home visits were arranged 1 month after discharge to assess and provide personalized guidance on medication adherence and daily care needs.

#### The intervention group underwent ACP in addition to routine care

1. Formation of the ACP intervention team. The team consisted of two oncologists, four oncology nurses, one psychologist, and one team coordinator. Doctors conducted condition assessment, provided disease-related information, and together with the nurses, communicated with patients and their primary caregivers to implement ACP interventions. The psychologist conducted psychological evaluations and provided counseling to address negative emotions and provide psychological support.
2. Team training. All team members completed 40 h of theoretical training on ACP. Team members were encouraged to participate in academic exchanges on palliative care. The psychologist provided scenario-based training on conducting family meetings, resolving family conflicts, and coordinating family opinions.
3. Through literature review, expert consultation and iterative revisions, an ACP protocol was developed. This process was combined with findings from qualitative interviews on facilitators and barriers to ACP participation among terminal cancer patients in our hospital, and the process was based on a trust-based behavioral change theory [19] as well as patterns of cognitive and emotional acceptance in patients [20].
4. Pilot study: Ten terminally ill cancer patients meeting the inclusion criteria and their primary caregivers were recruited for the pilot test. During implementation, identified issues were promptly addressed to further refine and optimize the intervention protocol.
5. Implementation of the care plan (Table 1). A one-on-one, face-to-face intervention approach was adopted. Each patient was assigned a dedicated oncology nurse for case management to ensure that communication took place when the patient was cognitively stable and receptive.

**Table 1:** Comparison of general information for patients and primary caregivers with terminal cancer in both groups

Individuals	Characteristic	Intervention group *N* = 30	Control group *N* = 30	χ^2^/t value	*p* value
Patients	Sex			0.06	0.79
	Male	16	15		
	Female	14	15		
	Marital status			0.07	0.97
	Married	24	23		
	Unmarried	2	2		
	Widowed	2	2		
	Divorced	2	3		
	Education level			0.22	0.97
	Primary school or below	17	18		
	Secondary school	12	11		
	Junior college or above	1	1		
	Religious belief			0.19	0.67
	Christianity	4	2		
	None	26	28		
	Payment method			0.63	0.73
	Cooperative medical insurance	9	7		
	Health insurance	8	7		
	Health insurance + cooperative	13	16		
	Residence status			0	1
	Lives alone	17	18		
	Lives with family	12	11		
	Diagnoses			0.67	0.88
	Lung cancer	11	13		
	Intestinal cancer	7	6		
	Liver cancer	5	6		
	Other	7	5		
	Monthly household income (CNY)			0.69	0.71
	< 2000	7	6		
	2000–5000	15	13		
	> 5000	8	11		
	Age (years), mean (SD)	50.90 (± 13.08)	52.93 (± 12.96)	0.61	0.55
	Diagnosis time (months), mean (SD)	23.57 (± 10.89)	22.00 (± 10.24)	0.57	0.57
Individuals	Characteristic	Intervention group *N* = 30	Control group *N* = 30	*χ*^*2*^*/t* value	*p* value
Primary caregivers	Sex			0	1
	Male	10	10		
	Female	20	20		
	Relationship with patient			0.38	0.94
	Spouse	16	14		
	Child	10	11		
	Parent	2	3		
	Other	2	2		
	Education level			0.61	0.74
	Primary school or below	10	8		
	Secondary school	15	18		
	Junior college or above	5	4		
	Age (years), mean (SD)	42.70 (± 11.82)	45.43 (± 11.65)	0.90	0.37

*SD *Standard deviation

**Fig. 1:**
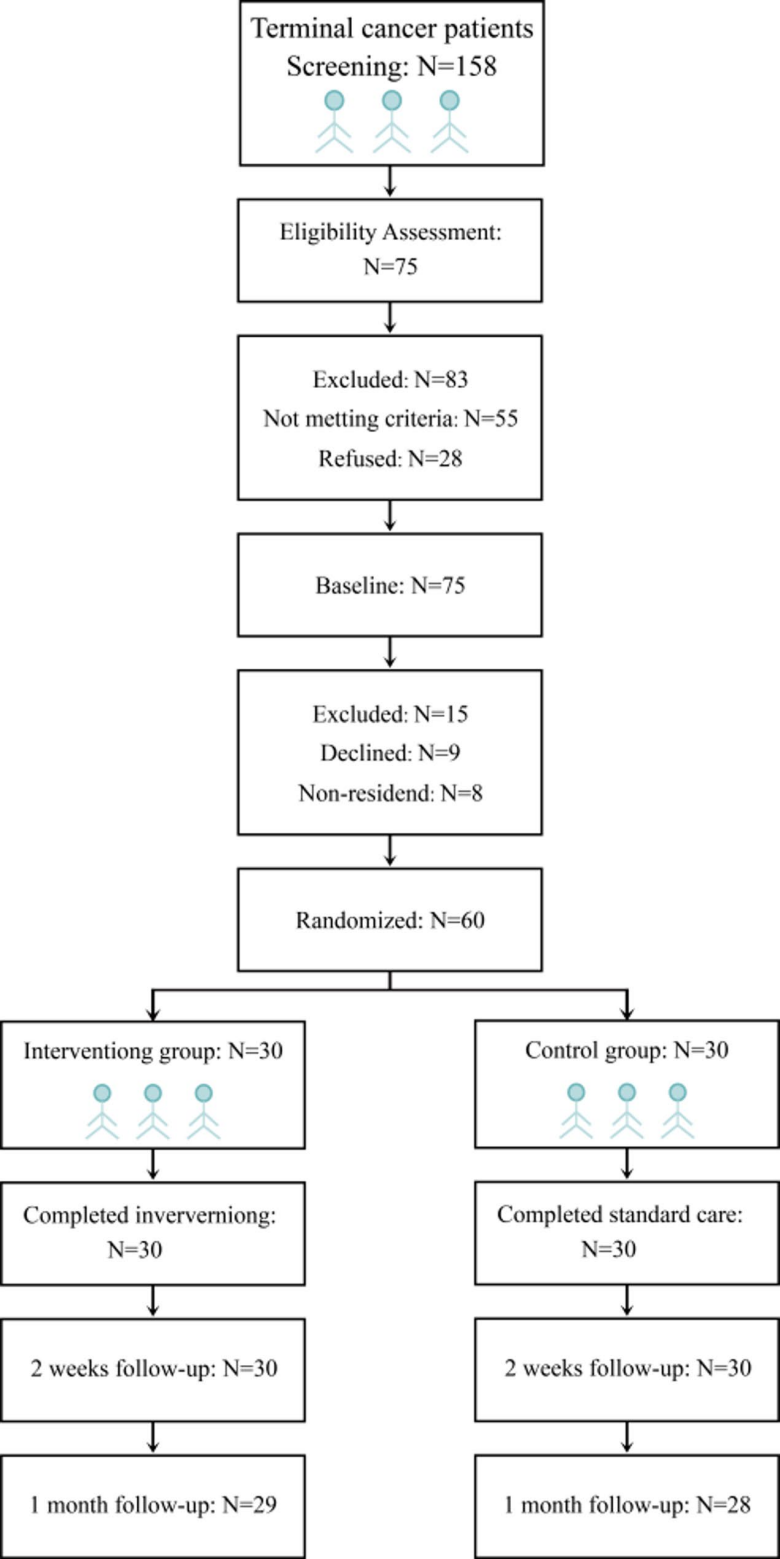
Research protocol

(i) Days 1–2. Conducting a comprehensive assessment to understand the patient’s family structure, functional status, interpersonal relationships, financial situation, customs, key decision-makers, educational levels of the patient and decision-makers, trust in medical services, and coping mechanisms. Personalized communication plans and content were developed based on these assessments.
(ii) Days 3–4. Establishing a good and trustworthy relationship with the patient and family decision-maker through life review [21] to understand their values, life perspectives, and beliefs.
(iii) Days 5–7. The decision-making aids [22] were used, including department-prepared educational materials and videos on various treatment options (e.g., chemotherapy, brachytherapy, interventional surgery) and procedures (e.g., cardiopulmonary resuscitation, intubation, mechanical ventilation, continuous renal replacement therapy). These materials objectively presented the pros and cons of each option and their corresponding outcomes. They were provided to patients and their primary caregivers, especially decision-makers, to facilitate targeted discussions and aid decision-making regarding ACP content.
(iv) Days 8–9. Oncology nurses conducted semi-structured interviews to explore the preferences of patients’ and primary caregivers’ (especially decision-makers). This process aimed to understand patients’ end-of-life care wishes and confirm their feelings and concerns.
(v) Days 10–11. Patients were guided to express their medical and care preferences, and any questions from them or their caregivers were addressed. Diandian Luo’s “Five Wishes” framework [23] was used to help patients to clarify their wishes about medical services, life-support treatments, how they wish to be treated by others, messages for family and friends, and preferred assistants. Based on each patient’s preferences, a tailored care plan was created, with flexibility to adjust it as needed.
(vi) Days 12–13. A family meeting was conducted, involving one doctor, one nurse, and the psychologist, lasting no more than 60 min. The family meeting encouraged patients and caregivers to share their illness experiences, express concerns and fears, discuss the care plan, and if necessary, designate a medical decision-making proxy. Respecting the patient’s end-of-life care wishes.
(vii) Follow-up. From day 14 onward, conducting of conducted home visits by oncology nurses to guide and assist with family care, address concerns raised by patients or decision-makers, and re-evaluate patient preferences for medical care. Making adjustments based on the patient’s needs and wishes. Weekly communication was continued through video or phone calls, or home visits, to provide further support and address questions. If the patient was re-admitted, the designated oncology nurse followed up with case tracking and provided comprehensive, continuous, and coordinated care (Fig. 1).

Throughout the entire process, healthcare providers gradually implemented intervention, allowing patients and caregivers to take the lead, while the providers acted as facilitators, coordinators, and guides.

### Evaluation indicators

#### Psychological status

We used two scales recommend by World Health Organization: the Generalized Anxiety Disorder-7 (GAD-7) scale [24] and Patient Health Questionnaire-9 (PHQ-9) [25].

The GAD-7 consists of seven items designed to assess anxiety symptoms over the past 2 weeks. Each item is scored from 0 to 3, corresponding to “Not at all,” “Several days,” “More than half the days,” and “Nearly every day,” respectively. The total score ranges from 0 to 21 and is interpreted as follows: 0–4 indicates no anxiety, 5–9 mild anxiety, 10–13 moderate anxiety, 14–18 moderately severe anxiety, 19–21 severe anxiety.

The PHQ-9 consists of nine items assessing depressive symptoms over the past week. Each item is scored similarly to the GAD-7, resulting in a total score ranging from 0 to 27. The total score is interpreted as follows: 0–4 indicates no depression, 5–9 mild depression, 10–14 moderate depression, 15–19 moderately severe depression, and 20–27 severe depression.

#### Quality of life

We used the EORTC QLQ-C30, a quality of life scale developed by the European Organization for Research and Treatment of Cancer specifically for cancer patients [26]. This scale consists of 30 items across 15 dimensions: one global health status item, five functional domains (physical, role, emotional, cognitive, social functioning), and nine symptom domains (fatigue, pain, nausea/vomiting, dyspnea, appetite loss, insomnia, constipation, diarrhea, and financial difficulties). Each domain is standardized on a scale from 0 to 100, higher scores in global health status and functional domains indicate better quality of life, while higher scores in symptom domains indicate worse quality of life.

#### Number of intensive care unit (ICU) admissions

The number of times patients were admitted to the ICU during the study period was recorded.

#### Length of hospital stay and expenses

The length of hospital stay and direct medical expenses incurred in the last 30 days before death were recorded.

### Quality control and data collection

Team members monitored and supervised each other throughout the study. The project leader was responsible for overall planning and ensuring the quality of intervention implementation. Issues encountered were promptly addressed through team discussions and training. After the data were collected, two people entered and verified the data to create a database.

Informed consent and baseline assessments for patients and their caregivers were completed within 2 days of admission. To evaluate the preliminary effects and potential issues of the intervention, as well as the stability of outcomes, we conducted follow-ups at two weeks and one month after the intervention to collect data on patient’s and primary caregivers’ psychological status, as well as patients’ quality of life. The number of ICU admissions, length of hospital stay, and expenses were retrospectively recorded after patient deaths.

### Statistical analysis

This study used EpiData 3.1 software for double data entry. After data validation, statistical analysis was performed using IBM SPSS 25.0. For deceased patients, data corresponding to 2 weeks post-intervention were used for intention-to-treat analysis. Normally distributed quantitative data were expressed as mean ± standard deviation, and compared between groups using the *t*-test. Non-normally distributed quantitative data were expressed as median and percentile, and the rank-sum test was used for inter-group comparisons. Qualitative data were described as percentage and the *χ*^*2*^ test was used for inter-group comparisons. Repeated measures analysis of variance (ANOVA) and simple effect analysis were used for comparisons of pre-intervention, 2 weeks post-intervention, and 1 month post-intervention data between the two groups. A *P*-value < 0.05 was considered to be statistically significant.

## Results

### Comparison of general information between groups

By the end of the study, two patients in the control group and one patient in the intervention group had died. No statistically significant differences in baseline characteristics were found between the two groups (*P* > 0.05, Table 2).

**Table 2:** Advance care planning (ACP) intervention for the experimental group

Phase	Duration	Key activities	Tools/techniques
Assessment	Days 1–2	Evaluate family dynamics, socioeconomic factors, healthcare literacy, trust	Customized assessment forms
Trust establishment	Days 3–4	Build rapport; explore values/beliefs via Life Review^[8]^	Narrative therapy techniques
Decision education	Days 5–7	Present treatment options (chemotherapy/CPR) using videos/handbooks	Decision aids^[9]^
Preference documentation	Days 8–11	Elicit care preferences using Five Wishes^[10]^; draft personalized ACP plan	Semi-structured interviews
Family consensus	Days 12–13	One-hour multidisciplinary family meeting to finalize proxy and care plan	Conflict resolution protocols
Follow-up	Week 4+	Home visits and weekly teleconsultations; inpatient case tracking for plan updates	

### Comparison of GAD-7 and PHQ-9 scores among patients and primary caregivers before and after intervention (2 weeks and 1 month) in both groups

Statistical analysis revealed that the ACP intervention significantly reduced anxiety symptoms among patients, as measured by GAD-7. The ACP group exhibited substantially lower anxiety than the control group throughout the study (*F*^*a*^(1,58) = 18.79, *p* < 0.001). More importantly, the pattern of anxiety reduction over time differed substantially between the two groups, as indicated by a significant group × time interaction effect (*F*^*a*^(2,116) = 8.02, *p* = 0.001). Follow-up simple effects analysis clarified that these between-group differences became particularly prominent at the 1-month follow-up, with the ACP group demonstrating significantly greater improvement compared to the control group (*p* < 0.001, Table 3.).

**Table 3.** Comparison of GAD-7 scores among patients and primary caregivers before and after intervention (2 weeks and 1 month) in both groups

Individuals	Group	N	Pre-I	Post-intervention		Time effect	Group effect	Interaction effect
				2 W Post-I	1 M Post-I			
Patients	Intervention group	30	14.20 ± 3.76	12.40 ± 3.52	10.33 ± 3.18	*F*^*a*^ = 2.95	*F*^*a*^ = 18.79	*F*^*a*^ = 8.02
	Control group	30	13.40 ± 3.33	14.63 ± 2.74	14.43 ± 2.62	*P* = 0.06	*P* < 0.001	*P* = 0.001
	*F*^*b*^ value		2.41	1.48	128.77			
	*p* value		0.13	0.23	< 0.001			

Individuals	Group	N	Pre-I	Post-intervention		Time effect	Group effect	Interaction effect
				2 W Post-I	1 M Post-I			
Primary caregivers	Intervention group	30	13.37 ± 2.85	11.27 ± 2.56	9.97 ± 2.22	*F*^*a*^ = 7.09	*F*^*a*^ = 35.91	*F*^*a*^ = 8.46
	Control group	30	13.90 ± 2.19	13.20 ± 2.55	14.20 ± 2.52	*P* = 0.001	*P* < 0.001	*P* < 0.001
	*F*^*b*^ value		0.66	8.59	47.57			
	*p* value		0.42	0.005	< 0.001			

“a” represents repeated measures analysis, and “b” represents simple effect analysis

A similar beneficial effect was observed on depressive symptoms (PHQ-9) in patients. Scores in the ACP group decreased significantly over time (*F*^*a*^(2,116) = 8.57, *p* < 0.001), and the extent of this reduction was significantly greater than that observed in the control group (*F*^*a*^(1,58) = 31.37, *p* < 0.001). The presence of a significant group × time interaction (*F*^*a*^(2,116) = 18.57, *p* < 0.001) confirmed that the trajectory of symptom improvement differed between the interventions. Simple effects analysis pinpointed that the ACP group’s superior improvement in depression was statistically significant at the 1-month after the intervention (*p* < 0.001, see Table 4.).

**Table 4.** Comparison of PHQ-9 scores among patients and primary caregivers before and after intervention (2 weeks and 1 month) in both groups

Individuals	Group	N	Pre-I	Post-intervention		Time effect	Group effect	Interaction effect
				2 W Post-I	1 M Post-I			
Patients	Intervention group	30	15.33 ± 4.12	11.10 ± 3.96	8.63 ± 3.94	*F*^*a*^ = 8.57	*F*^*a*^ = 31.37	*F*^*a*^ = 18.57
	Control group	30	15.03 ± 3.79	12.50 ± 4.90	17.73 ± 5.25	*P* < 0.001	*P* < 0.001	*P* < 0.001
	*F*^*b*^ value *p* value		0.09	1.48	57.60			
			0.77	0.23	< 0.001			

Individuals	Group	N	Pre-I	Post-intervention		Time effect	Group effect	Interaction effect
				2 W Post-I	1 M Post-I			
Primary caregivers	Intervention group	30	16.13 ± 5.19	14.93 ± 4.01	9.43 ± 2.62	*F*^*a*^ = 22.16	*F*^*a*^ = 10.20	*F*^*a*^ = 13.33
	Control group	30	14.00 ± 3.33	17.87 ± 2.62	14.30 ± 4.73	*P* < 0.001	*P* = 0.002	*P* < 0.001
	*F*^*b*^ value *p* value		3.58	11.25	24.31			
			0.06	0.001	< 0.001			

“a” represents repeated measures analysis, and “b” represents simple effect analysis

Notably, the positive effects of the ACP intervention also extended to the primary caregivers. Caregivers in the ACP group reported a significant reduction in their own anxiety (GAD-7) over the course of the study (*F*^*a*^(2,116) = 7.09, *p* = 0.001), and their outcomes were significantly better than those of caregivers in the control group (*F*^*a*^(1,58) = 35.91, *p* < 0.001). A significant group × time interaction (*F*^*a*^(2,116) = 8.46, *p* < 0.001) indicated that the trajectory of anxiety reduction for caregivers also differed by group. Subsequent simple effects analysis confirmed that the reduction in caregiver anxiety was significantly greater in the ACP group at both the 2-week and 1-month follow-ups after the intervention (*p* < 0.001, Table 3.).

Depressive symptoms (PHQ-9) among primary caregivers in the ACP group demonstrated a significant reduction over the study period (*F*^*a*^(2,116) = 22.16, *p* < 0.001), with markedly greater improvement compared to caregivers in the control group (*F*^*a*^(1,58) = 10.20, *p* = 0.002). A significant group × time interaction was observed (*F*^*a*^(2,116) = 13.33, *p* < 0.001), indicating that the trajectory of symptom improvement differed substantially between the two groups. Subsequent simple effects analysis confirmed that the reduction in caregiver depression was significantly greater in the ACP group at both the 2-week and 1-month follow-ups after the intervention. (*p* < 0.001, Table 4.).

### Comparison of overall quality of life scores and dimension scores among patients after intervention in both groups

#### Comparison of global health status scores between patients in both groups

Patients in the ACP group demonstrated significant improvement in global health-related quality of life over the course of the study (*F*^*a*^(1.67, 96.87) = 4.37, *p* = 0.021), with overall outcomes being significantly better than those observed in the control group (*F*^*a*^(1, 58) = 15.88, *p* < 0.001). A significant group × time interaction effect was found (*F*^*a*^(1.67, 96.87) = 13.22, *p* < 0.001), indicating that the pattern of improvement differed between the two groups. Simple effects analysis confirmed that the ACP group showed significantly greater improvement in quality of life compared to the control group specifically at both the 2-week and 1-month follow-ups after the intervention (*p* < 0.001, Table 5).

**Table 5:** Comparison of global health status scores among patients before and after intervention in both groups

Group	*N*	Pre-I	Post-intervention		Time effect	Group effect	Interaction effect
			2 W Post-I	1 M Post-I			
Intervention group	30	44.44 ± 20.09	48.33 ± 12.46	49.99 ± 14.84	*F*^*a*^ = 4.37	*F*^*a*^ = 15.88	*F*^*a*^ = 13.22
Control group	30	50.27 ± 14.60	37.50 ± 10.89	29.44 ± 10.43	*P* = 0.021	*P* < 0.001	*P* < 0.001
*F*^*b*^ value *p* value		1.66	12.87	38.51			
		0.203	0.001	< 0.001			

“a” represents repeated measures analysis, and “b” represents simple effect analysis

#### Comparison of scores for the five quality of life functional domains between patients in both groups

Patients in the ACP group demonstrated significant improvement in physical function over time [*F*^*a*^(2,116) = 16.59, *p* < 0.05], with scores being significantly higher than those in the control group [*F*^*a*^(1,58) = 28.28, *p* < 0.05]. A significant group × time interaction was observed [*F*^*a*^(2,116) = 55.49, *p* < 0.05], indicating different trajectories between the two groups. Simple effects analysis confirmed that the ACP group showed significantly better physical function than the control group at the 1-month after the intervention (*p* < 0.05).

A significant group × time interaction was found for role function [*F*^*a*^(2,116) = 34.62, *p* < 0.05], reflecting divergent patterns of change between groups. At 2 weeks post-intervention, the ACP group had significantly lower scores than the control group (*p* < 0.05); however, by the 1-month assessment, this pattern reversed, with the ACP group showing significantly higher role functioning scores (*p* < 0.05).

Emotional function improved significantly over time in the ACP group [*F*^*a*^(2,116) = 8.08, *p* < 0.05], with overall scores being significantly superior to the control group [*F*^*a*^(1,58) = 28.32, *p* < 0.05]. A significant group × time interaction was observed [*F*^*a*^(2,116) = 27.49, *p* < 0.05], indicating differing rates of improvement. Simple effects analysis confirmed significantly better emotional functioning in the ACP group at 1 month after the intervention (*p* < 0.05).

For cognitive function, a significant group difference was observed [*F*^*a*^(1,58) = 14.61, *p* < 0.05], accompanied by a significant group × time interaction [*F*^*a*^(2,116) = 22.34, *p* < 0.05]. Simple effects analysis revealed that the ACP group demonstrated significantly better cognitive function than the control group at the 1-month follow-up (*p* < 0.05).

A significant improvement was also evident in social function scores [*F*^*a*^(2,116) = 33.23, *p* < 0.05]. Simple effects analysis confirmed that the ACP group showed significantly better social functioning compared to the control group at the 1-month assessment (*p* < 0.05, Tables 6. and 7).

**Table 6.** Repeated measures ANOVA for five functional dimensions of quality of life before and after intervention in both groups

Group		N	Pre-I	Post-intervention		Time effect	Group effect	Interaction effect
				2 W Post-I	1 M Post-I			
Physical function	Intervention group	30	60.66 ± 14.15	60.44 ± 13.44	70.43 ± 12.32	F^*^ = 16.59	F^*^ = 28.28	F^*^ = 55.49
	Control group	30	65.77 ± 11.17	55.55 ± 17.45	29.99 ± 15.14			
Role function	Intervention group	30	61.11 ± 14.07	57.78 ± 19.44	77.21 ± 11.14	*F* = 0.86	*F* = 1.41	F^*^ = 34.62
	Control group	30	66.09 ± 16.95	74.13 ± 13.79	47.12 ± 18.93			
Emotional function	Intervention group	30	37.22 ± 24.44	45.83 ± 18.79	73.61 ± 19.46	F^*^ = 8.08	F^*^ = 28.32	F^*^ = 27.49
	Control group	30	36.94 ± 23.02	39.44 ± 18.56	29.44 ± 16.91			
Cognitive function	Intervention group	30	47.22 ± 15.83	52.77 ± 18.09	70.55 ± 14.96	*F* = 1.58	F^*^ = 14.61	F^*^ = 22.34
	Control group	30	54.99 ± 21.51	47.22 ± 10.80	39.44 ± 15.47			
Social function	Intervention group	30	31.66 ± 14.08	37.77 ± 14.49	47.22 ± 16.99	*F* = 4.15	*F* = 5.63	F^*^ = 33.23
	Control group	30	37.77 ± 14.47	41.39 ± 16.74	18.88 ± 9.77			

*: *P* < 0.05

**Table 7:** Simple effect analysis for five functional dimensions of quality of life before and after intervention in both groups

			*N*	Intervention	Control	F
Physical function	Pre-I		30	60.66 ± 14.15	65.77 ± 11.17	2.41
	Post-intervention	2 W Post-I	30	60.44 ± 13.44	55.55±17.45	1.48
		1 M Post-I	30	70.43 ± 12.32	29.99±15.14	128.77^*^
Role function	Pre-I		30	61.11 ± 14.07	66.09±16.95	1.51
	Post-intervention	2 W Post-I	30	57.78 ± 19.44	74.13±13.79	13.81^*^
		1 M Post-I	30	77.21 ± 11.14	47.12±18.93	55.81^*^
Emotional function	Pre-I		30	37.22 ± 24.44	36.94±23.02	0.002
	Post-intervention	2 W Post-I	30	45.83 ± 18.79	39.44±18.56	1.76
		1 M Post-I	30	73.61 ± 19.46	29.44±16.91	88.02^*^
Cognitive function	Pre-I		30	47.22 ± 15.83	54.99 ± 21.51	2.54
	Post-intervention	2 W Post-I	30	52.77 ± 18.09	47.22 ± 10.80	2.09
		1 M Post-I	30	70.55 ± 14.96	39.44±15.47	62.72^*^
Social function	Pre-I		30	31.66 ± 14.08	37.77±14.47	4.15
	Post-intervention	2 W Post-I	30	37.77 ± 14.49	41.39±16.74	5.63
		1 M Post-I	30	47.22 ± 16.99	18.88±9.77	33.23^*^

Note: *: *p* < 0.05

#### Comparison of scores in the nine symptom domains for quality of life between patients in both groups

A significant group × time interaction was observed for fatigue symptoms [*F*^*a*^(1.73,100.31) = 22.48, *p* < 0.05], indicating that the pattern of fatigue change differed between groups over time. Simple effects analysis confirmed that the ACP group reported significantly less fatigue than the control group at the 1-month follow-up (*p* < 0.05).

For nausea and vomiting symptoms, a significant group difference was found [*F*^*a*^(1,58) = 14.25, *p* < 0.05], along with a significant group × time interaction [*F*^*a*^(2,116) = 15.68, *p* < 0.05]. Simple effects analysis showed that the ACP group experienced significantly less nausea and vomiting than the control group at both the 2-week and 1-month assessments (*p* < 0.05).

Pain symptoms improved significantly over time in the ACP group [*F*^*a*^(2,116) = 11.36, *p* < 0.05], with overall lower pain levels than the control group [*F*^*a*^(1,58) = 13.57, *p* < 0.05]. A significant group × time interaction [*F*^*a*^(2,116) = 20.13, *p* < 0.05] indicated different trajectories of pain improvement. Simple effects analysis revealed significantly better pain control in the ACP group at both the 2-week and 1-month follow-up time points (*p* < 0.05).

A significant group difference was observed in dyspnea scores [*F*^*a*^(1,58) = 12.70, *p* < 0.05], with a significant group × time interaction [*F*^*a*^(2,116) = 9.38, *p* < 0.05]. Simple effects analysis confirmed that the ACP group reported significantly less shortness of breath than the control group at both follow-up time points (*p* < 0.05).

Insomnia symptoms improved significantly over time in the ACP group [*F*^*a*^(2,116) = 8.27, *p* < 0.05], with overall better sleep quality than the control group [*F*^*a*^(1,58) = 14.31,*p* < 0.05]. A significant group × time interaction [*F*^*a*^(2,116) = 14.46, *p* < 0.05] indicated different patterns of improvement. Simple effects analysis showed significantly better sleep outcomes in the ACP group at the 1-month assessment (*p* < 0.05).

A significant group × time interaction was found for appetite loss [*F*^*a*^(2,116) = 6.02, *p* < 0.05], with simple effects analysis confirming that the ACP group showed significantly less appetite loss than the control group at the 1-month assessment (*p* < 0.05).

For constipation symptoms, a significant group × time interaction was observed [*F*^*a*^(2,116) = 29.62, *p* < 0.05]. Simple effects analysis revealed that the ACP group experienced significantly less constipation than the control group at both the 2-week and 1-month assessments (*p* < 0.05).

A significant group difference was found in diarrhea scores [*F*^*a*^(1,58) = 22.14, *p* < 0.05], along with a significant group × time interaction [*F*^*a*^(2,116) = 16.41, *p* < 0.05]. Simple effects analysis confirmed that the ACP group had significantly better diarrhea control than the control group at both follow-up assessments (*p* < 0.05).

A significant group × time interaction was observed for financial distress [*F*^*a*^(2,116) = 30.37, *p* < 0.05]. Simple effects analysis showed that the ACP group reported significantly less financial distress than the control group at the 1-month post-intervention (*p* < 0.05, Tables 8. and 9).

**Table 8.** Repeated measures ANOVA for nine symptom dimensions of quality of life before and after intervention in both groups

Group		N	Pre-I	Post-intervention		Time effect	Group effect	Interaction effect
				2 W Post-I	1 M Post-I			
Fatigue	Intervention group	30	60.36 ± 15.89	55.55 ± 16.76	42.22 ± 13.18	*F* = 3.24	*F* = 3.26	F^*^ = 22.48
	Control group	30	57.77 ± 15.82	49.25 ± 21.38	68.14 ± 17.44			
Nausea/vomiting	Intervention group	30	53.33 ± 14.78	48.33 ± 17.70	36.67 ± 14.11	*F* = 1.38	F^*^ = 14.25	F^*^ = 15.68
	Control group	30	48.33 ± 17.70	60.00 ± 16.72	62.78 ± 18.92			
Pain	Intervention group	30	58.89 ± 18.94	35.56 ± 14.99	32.22 ± 16.34	F^*^ = 11.36	F^*^ = 13.57	F^*^ = 20.13
	Control group	30	49.99 ± 15.78	44.99 ± 17.04	61.11 ± 17.69			
Dyspnea	Intervention group	30	48.88 ± 22.71	31.11 ± 27.59	26.66 ± 23.81	*F* = 2.22	F^*^ = 12.70	F^*^ = 9.38
	Control group	30	44.44 ± 23.70	44.44 ± 20.22	58.88 ± 25.79			
Insomnia	Intervention group	30	59.99 ± 26.84	34.44 ± 23.95	24.44 ± 21.32	F^*^ = 8.27	F^*^ = 14.31	F^*^ = 14.46
	Control group	30	53.33 ± 24.13	47.77 ± 20.87	62.22 ± 25.87			
Appetite loss	Intervention group	30	57.77 ± 26.16	45.55 ± 20.49	45.55 ± 22.29	*F* = 1.30	*F* = 1.73	F^*^ = 6.02
	Control group	30	51.11 ± 20.96	53.33 ± 18.77	62.22 ± 28.68			
Constipation	Intervention group	30	56.66 ± 31.74	55.55 ± 29.47	25.55 ± 20.87	*F* = 3.38	*F* = 0.97	F^*^ = 29.62
	Control group	30	51.11 ± 27.31	39.99 ± 25.37	63.33 ± 28.16			
Diarrhea	Intervention group	30	36.66 ± 25.29	28.89 ± 24.34	17.78 ± 16.91	*F* = 1.28	F^*^ = 22.14	F^*^ = 16.41
	Control group	30	34.44 ± 22.29	54.44 ± 28.34	58.89 ± 28.61			
Financial difficulties	Intervention group	30	59.99 ± 29.56	48.89 ± 31.24	17.78 ± 19.04	*F* = 3.90	*F* = 3.904	F^*^ = 30.37
	Control group	30	47.77 ± 24.26	49.99 ± 27.33	61.11 ± 27.79			

*: *P* < 0.05

**Table 9:** Simple effects analysis for nine symptom dimensions of quality of life before and after intervention in both groups

			*N*	Intervention	Control	F
Fatigue	Pre-I		30	60.36 ± 15.89	57.77 ± 15.82	0.40
	Post-intervention	2 W Post-I	30	55.55 ± 16.76	49.25±21.38	1.61
		1 M Post-I	30	42.22 ± 13.18	68.14±17.44	42.19^*^
Nausea/Vomiting	Pre-I		30	53.33 ± 14.78	48.33±17.70	1.41
	Post-intervention	2 W Post-I	30	48.33 ± 17.70	60.00±16.72	6.89^*^
		1 M Post-I	30	36.67 ± 14.11	62.78±18.92	36.71^*^
Pain	Pre-I		30	58.89 ± 18.94	49.99±15.78	3.89
	Post-intervention	2 W Post-I	30	35.56 ± 14.99	44.99±17.04	5.19^*^
		1 M Post-I	30	32.22 ± 16.34	61.11±17.69	43.19^*^
Dyspnea	Pre-I		30	48.88 ± 22.71	44.44±23.70	0.55
	Post-intervention	2 W Post-I	30	31.11 ± 27.59	44.44±20.22	4.56^*^
		1 M Post-I	30	26.66 ± 23.81	58.88±25.79	25.27^*^
Insomnia	Pre-I		30	59.99 ± 26.84	53.33±24.13	1.02
	Post-intervention	2 W Post-I	30	34.44 ± 23.95	47.77±20.87	5.29
		1 M Post-I	30	24.44 ± 21.32	62.22±25.87	38.09^*^
Appetite loss	Pre-I		30	57.77 ± 26.16	51.11±20.96	1.19
	Post-intervention	2 W Post-I	30	45.55 ± 20.49	53.33±18.77	2.35
		1 M Post-I	30	45.55 ± 22.29	62.22±28.68	6.32^*^
Constipation	Pre-I		30	56.66 ± 31.74	51.11±27.31	0.53
	Post-intervention	2 W Post-I	30	55.55 ± 29.47	39.99±25.37	4.80^*^
		1 M Post-I	30	25.55 ± 20.87	63.33±28.16	34.85^*^
Diarrhea	Pre-I		30	36.66 ± 25.29	34.44±22.29	0.13
	Post-intervention	2 W Post-I	30	28.89 ± 24.34	54.44±28.34	14.04^*^
		1 M Post-I	30	17.78 ± 16.91	58.89±28.61	45.89^*^
Financial difficulties	Pre-I		30	59.99 ± 29.56	47.77±24.26	3.07
	Post-intervention	2 W Post-I	30	48.89 ± 31.24	49.99±27.33	0.02
		1 M Post-I	30	17.78 ± 19.04	61.11±27.79	49.61^*^

*: *P* < 0.05

### Comparison of the number of ICU admissions between patients in both groups after intervention

In the intervention group, 29 patients (96.67%) had no ICU admissions, one patient (3.33%) had one ICU admission, and none had two admissions. In the control group, 18 patients (60%) had no ICU admissions, 10 patients (33.3%) had one ICU admission, and two patients (6.67%) had two ICU admissions. A statistically significant difference was observed in the number of ICU admissions between the two groups (*χ*^*2*^ = 13.92, *P* < 0.05), indicating that the control group had more ICU admissions than the intervention group.

### Comparison of length of hospital stay and direct medical costs in the 30 days before death between patients in both groups

In the intervention group, the median length of hospital stay in the 30 days before death was 4 days (interquartile range: 3–5); in contrast, in the control group, it was 10.5 days (interquartile range: 7–18.25). The overall distribution of length of hospital stay in the 30 days before death showed a statistically significant difference between the two groups (*Z* = 5.37, *P* < 0.05), with the control group having a longer length of hospital stay than the intervention group.

The median direct medical cost in the 30 days before death was RMB 4901.50 (interquartile range: 3929.75–6810.75) for the intervention group, compared to RMB 18,226 (interquartile range: 12,047–26,531.25) for the control group. The overall distribution of direct medical costs in the 30 days before death also showed a statistically significant difference between the two groups (*Z* = 5.99, *P* < 0.05), indicating that the control group incurring higher costs than the intervention group.

## Discussion

### ACP reduces anxiety and depression in patients with terminal cancer and their caregivers

Patients with terminal cancer often experience negative emotions such as anxiety and depression due to various cancer symptoms, side effects of treatment, fear of the unknown associated with the disease, and feelings of guilt or reluctance to leave their families. The primary caregivers of these patients’ are also prone to pessimism and depression owing to prolonged and intense caregiving responsibilities. Reducing the suffering of patients and their families is one of the main goals of palliative care [27].

The results of this study showed that before the intervention, both groups of patients and their primary caregivers experienced varying degrees of anxiety and depression; however, there were no statistically significant differences between the groups. Two weeks after the intervention, there were no significant differences in the anxiety and depression levels of patients. In contrast, at 2 weeks and 1 month post-intervention, significant differences were observed in the anxiety and depression levels of primary caregivers between the two groups. At 1 month post-intervention, patients’ anxiety and depression levels also showed significant differences.

These findings may be attributed to the following factors. After 2 weeks of ACP implementation, patients often experienced uncertainty about decision-making, including whether to withdraw or forgo certain medical interventions, which can be a challenging decision for patients with cancer [28]. ACP encourages open discussions between patients and their primary caregivers through family meetings and similar formats, which facilitates emotional communication and interaction. This provides a means for psychological relief and emotional support [29]. By clarifying future treatment plans, ACP can reduce the stress associated with medical decision-making for both patients and their caregivers and improve family relationships [30]. Life review interviews and personalized communication can further alleviate negative emotions in patients and their primary caregivers. The implications of our study results align with this evidence.

During the early stages of ACP intervention, it is crucial to identify patient decision-making needs and address conflicts promptly. Additionally, providing comprehensive informational support and emotional assistance is also essential.

### ACP improves quality of life for patients with terminal cancer

Quality of life is considered a comprehensive measure of an individual’s or group’s perceived physical, psychological, and social well-being [31]. For patients with cancer, physical symptoms caused by the disease and side effects of treatment severely affect their quality of life. The results of our study showed that there were no significant differences between the two groups in the four functional domains (physical, emotional, social, and cognitive) or in symptoms such as fatigue, appetite loss, and financial difficulties at 2 weeks post-intervention. Analysis indicated that after 2 weeks of ACP implementation, patients experienced negative emotions stemming from decision-making confusion. Additionally, the symptom burden during treatment breaks caused physiological stress that hindered the recovery of physical functions, leading to impaired functional domains. Patients also experienced moderate anxiety and depression, which in turn affected their appetite [32].

Fatigue is one of the most severe and frequent symptoms during treatment breaks [33]. Additionally the high costs of early-stage treatment can lead to patients experiencing considerable financial stress. At 2 weeks post-intervention, significant differences were observed between the two groups in the scores for global health status, role functioning, pain, nausea/vomiting, dyspnea, insomnia, constipation, and diarrhea domains. At 1 month post-intervention, statistically significant differences were found across all quality-of-life domains between the groups. Analysis indicated that our study guided patients in expressing their treatment preferences and gave them the opportunity to exercise autonomy in decision-making. Patients, caregivers, and health care professionals engaged in effective communication. Video-assisted tools helped patients and caregivers fully understand various treatment methods. This leads patients to prefer palliative care to alleviate physical symptoms and reduce suffering, while avoiding aggressive treatments.

Family meetings allowed patients and their caregivers to reach a consensus on end-of-life care preferences and facilitated timely adjustment to goals [34]. Patients were able to effectively plan their subsequent work and life [35]. Caregivers encouraged them to participate in preferred social and recreational activities, which reduced physical discomfort and improved quality of life.

The implications of our study are that during the early stages of ACP intervention, methods such as psychological education, physical activity interventions, Traditional Chinese Medicine, and pharmaceutical interventions should be used to accelerate recovery in patients’ functional domains. Spiritual intervention [36] should be provided to encourage patients to openly disclose their feelings [37], thereby facilitating targeted psychological support.

### ACP reduces the number of ICU admissions, length of hospital stay, and medical costs

Traditional Confucian cultural values, such as “reverence for life and avoidance of death” and “filial piety,” influence patients with terminal cancer in China [38]. As a result, they often receive invasive or life-prolonging treatments at the end of life, including cardiopulmonary resuscitation, mechanical ventilation, dialysis, intubation, and blood transfusions [39]. These high-intensity treatments increase the likelihood of ICU admission and substantially drive up hospitalization costs. Additionally, there is a positive correlation between length of hospital stay and hospitalization costs for patients with terminal cancer [40].

This study showed that the control group had a higher number of ICU admissions compared to the intervention group. Moreover, the control group experienced significantly longer hospital stays and higher direct medical costs in the 30 days before death. All these differences were statistically significant. Studies have shown that ACP is an effective tool for controlling hospitalization costs and optimizing resource utilization [41], helping to prevent resource waste caused by overtreatment [42].

Our study indicated that efforts should be made to strengthen the promotion of ACP education, improve ACP-related legislation, and encourage broader acceptance and participation in ACP, thereby reducing waste and addressing the unequal distribution of medical resources.

### Limitations and future directions

The inclusion criterion requiring primary caregivers to provide continuous care for over 72 h may have excluded patients with inadequate social support, such as those lacking alternative caregivers, thereby introducing selection bias. As this was a single-center study, geographic bias might exist in the findings due to regional cultural variations in the acceptance of ACP. Additionally, the small sample size and short follow-up period may affect the generalization of the results to a heterogeneous group of end-stage patients.

Although ACP demonstrated clinical benefits in this study, its resource feasibility for implementation in primary hospitals warrants careful evaluation. Effective ACP delivery necessitates dedicated leadership from physicians and nurses. This requirement presents significant challenges for resource-constrained primary care teams operating in clinically demanding environments. Furthermore, the absence of dedicated reimbursement mechanisms for ACP services within current healthcare policies may impede their long-term scalability and sustainability.

Future research should prioritize establishing an ACP service delivery framework tailored to China’s sociocultural context. Strategic emphasis should be placed on four priorities: (1) Innovating intervention models by developing evidence-based, cost-effective implementation methods, such as group-based facilitated discussions and digital health tools; (2) Enhancing public engagement through nationwide ACP awareness campaigns to overcome cultural barriers to end-of-life care planning; (3) Implementing systematic competency-building initiatives for healthcare professionals via standardized ACP communication skill training; (4) Establishing a multidisciplinary ACP governance model that integrates legal safeguards, ethical guidelines, and insurance reimbursement mechanisms to ensure clinical feasibility and sustainability [43].

## Conclusion

This study used a combined ACP intervention model that integrated the advantages of life review, video-assisted decision-making, semi-structured interviews, and family meetings. Additionally, the model incorporated comprehensive case management throughout the process. With adequate preparation, this ACP intervention model can be implemented smoothly for patients with terminal cancer in primary hospitals and has also shown preliminary intervention effectiveness in feasibility studies. The preliminary efficacy observed in this feasibility trial provides critical baseline data for subsequent large-scale investigations.

## Data Availability

The datasets used and analyzed during the current study are available from the corresponding author upon reasonable request.
